# Impact of mild hypo- and hyperventilation on cerebral oxygen supply during general anesthesia

**DOI:** 10.1186/s13741-025-00517-9

**Published:** 2025-03-17

**Authors:** Philipp Groene, Miriam Rapp, Tobias Ninke, Peter Conzen, Klaus Hofmann-Kiefer

**Affiliations:** Department of Anaesthesiology, LMU University Hospital, LMU Munich, Marchioninistr. 15, Munich, 81377 Germany

**Keywords:** Hypoventilation, Hyperventilation, Carbon dioxide, Regional cerebral oxygen saturation, Near-infrared spectroscopy

## Abstract

**Objective:**

Cerebral blood flow autoregulation is affected by several physiologic and medical factors. Especially arterial carbon dioxide pressures (PaCO_2_) impact cerebral blood flow. Only extensive changes in end-tidal CO_2_ have been studied so far. The aim of this study was to evaluate the impact of mild hypo- and hyperventilation on cerebral blood flow as assessed by regional cerebral red blood cell oxygen saturation (rSO_2_) in two age groups.

**Methods:**

Two groups of patients were compared under general anesthesia before the surgical procedure was started: A younger patient group (age < 40 years; YP) and older patients aged > 60 years (OP). Anesthetic management was standardized. In both groups, end-tidal CO_2_ was adjusted either to a low-normal value of 35–37 mmHg or a high-normal value of 43–45 mmHg for 15 min each. The sequence of these interventions was randomized. rSO_2_ was estimated by near-infrared spectroscopy (NIRS). The primary outcome was defined as the difference in rSO2 between hypo- and hyperventilation between the two age groups.

**Results:**

A total of 78 patients were included. In both groups, there was a statistically significant difference in rSO2 values after 15 min of hypo- versus hyperventilation. In the YP-group, rSO_2_ was 74 ± 4% after 15 min of hypoventilation and decreased to 68 ± 6% during hyperventilation (*p* < 0.001). In the OP-group, rSO_2_ was 71 ± 5% and 65 ± 6%, respectively (*p* < 0.001). There was no difference concerning changes in comparison of younger and elder patient groups (in both groups, Δ rSO_2_ = 6 ± 3%; *p* = 0.732).

**Conclusion:**

Even mild hypoventilation increased rSO_2_ compared to mild hyperventilation and this difference occurred independent of age.

## Introduction

As a particularly metabolically active organ, the brain requires a constant supply of energy, especially in the form of oxygen. Under physiologic conditions and at a constant metabolic rate, this is ensured by keeping cerebral blood flow at a constant level despite changes in cerebral perfusion pressure (CPP), a phenomenon known as cerebral autoregulation (Armstead 2016; Paulson et al. 1990; Silverman and Petersen 2022). However, autoregulation fails outside certain limits of perfusion pressure, and various drugs used during anesthesia (e.g., inhalative anesthetics and propofol) are known to affect autoregulation as well (Cole et al. 2007; Dagal and Lam 2009; Engelhard et al. 2001; Gupta et al. 1997; Robertson et al. 2022; Strebel et al. 1995; Summors et al. 1999). Therefore, care must be taken to ensure that CPP is always capable of maintaining a sufficient oxygen supply.

Cerebral blood flow autoregulation may be hampered not only by various pathologic but also by physiological factors (e.g., age); medical procedures (e.g., mechanical ventilation, general anesthesia); and drugs (e.g., inhalation anesthetics) (De Deyne et al. 2004; Meng and Gelb 2015; Paulson et al. 1990; Robertson et al. 2022; Silverman and Petersen 2022; Sponheim et al. 2003; Zhang et al. 2016).

In this context, arterial carbon dioxide pressure has been known for its vasoactive properties for decades (Ainslie and Duffin 2009; Gibbs et al. 1935). When arterial carbon dioxide pressure (PaCO_2_) decreases, the cerebral arterial resistance vessels react with constriction and vice versa (Ainslie and Duffin 2009; Gibbs et al. 1935; Kety and Schmidt 1946; Meng et al. 2012). However, PaCO_2_ not only influences vascular tone but also has an impact on autoregulation itself. For example, hypercapnia narrows the CPP range where blood flow remains constant (Meng and Gelb 2015). Due to this effect, a PaCO_2_ outside the normal range can significantly interfere with the brain’s oxygen supply.

During anesthesia, regional cerebral red blood cell oxygen saturation (rSO_2_) can be assessed by near-infrared spectroscopy (NIRS) (Jöbsis 1977). NIRS is widely used to evaluate sufficient cerebral oxygenation during surgery in critically ill patients. Examples of this are carotid surgery and cardiac surgery. The actual NIRS can provide information on cerebral oxygenation, but only further processing can provide information on cerebral autoregulation, then called cerebral oximetry index (COX) (Vu et al. 2024).

Previous research in the context of rSO_2_ as surrogate of cerebral oxygenation, however, has concentrated on quite extensive PaCO_2_ variations. The comparison of mild hypo- and hyperventilation and their effects on cerebral blood oxygen saturation has not been investigated, so far. In addition, the effect of age on rSO2 induced by CO_2_ variations within the physiological range remain unclear (Burkhart et al. 2011; Goettel et al. 2016).

Therefore, this study aimed to investigate the influence of mild hypoventilation (end-tidal CO_2_ 45 mmHg) or hyperventilation (end-tidal CO_2_ 35 mmHg) on rSO_2_ with a special focus on potential variations between younger and older patients.

## Methods

This prospective, observational, single-center study was conducted in years 2020 and 2021. The study protocol had been approved by the Ludwig-Maximilians-University ethics committee (No 19–920). The study was performed in accordance with the Declaration of Helsinki, with written informed consent being obtained from all participants before they undertook any of the study procedures. The study was registered in the German Clinical Trials Register (No.: DRKS00033414).

The measurements were done in the operating theatre before surgical procedures were started. Patients were in the supine position which was not changed during measurements. Two patient groups were formed: a younger age group (< 40 years; YP) and an older group of equal size aged > 60 years (OP). This classification was made with respect to the published work on the influence of age on cerebral autoregulation (Burkhart et al. 2011). Patients with arterial hypertension, chronic obstructive pulmonary disease (COPD), normal-pressure hydrocephalus, symptomatic stenosis of an internal carotid artery, history of stroke, or congestive heart failure were excluded. Anesthetic management was standardized in both groups. General anesthesia was induced according to in-house standards with propofol and sufentanil, supplemented by a muscle relaxant if necessary. Airway management was done by an endotracheal tube or larynx mask. Maintenance was done with propofol and repetitive doses of sufentanil as needed. Depth of anesthesia was monitored using the patient state index (PSI, Masimo Corporation, Irvine, USA) with a target value of 50–30. Decreases in blood pressure > 30% from its initial preanesthetic value were not tolerated and, if necessary, treated using norepinephrine. Norepinephrine was the only vasopressor used and blood pressure was measured using a cuff on the upper arm.

Mechanical ventilation was adjusted in accordance with the clinic’s standards: normofrequency (8–14 breaths per minute), tidal volume calculated for the patient’s ideal body weight (6–8 ml/kg), inspiratory oxygen fraction (FiO2, 0.4), and a normal-ranged end-tidal CO_2_ of 35–45 mmHg. These parameters were held constant for an equilibration period of at least 5 min (T0). Thereafter, end-tidal CO_2_ was either adjusted to a low-normal value (35–37 mmHg) or a high-normal value (43–45 mmHg) by adjusting the respiratory rate. The order of low or high end-tidal CO2 was randomized. This condition was then kept constant for 15 min.

Regional cerebral red blood cell oxygen saturation (rSO_2_) was measured using near-infrared spectroscopy (NIRS; MASIMO Corporation, Irvine, USA). The electrode was placed on the forehead as standard, according to the manufacturer’s instructions. Measurements of rSO_2_ were obtained at T0 and repeated at 5, 10, and 15 min after the desired end-tidal CO_2_ target values had been attained (T1-T3). Thereafter, the ventilatory rate was adjusted again to attain the second experimental step of either hypo- or hyperventilation within a few minutes. Measurements were then repeated as in the first period.

The primary outcome was defined as the difference in rSO_2_ between hypoventilation and hyperventilation in comparison of both age groups. Secondary outcomes were considered changes within each group.

## Statistical analysis

An a priori sample size calculation was performed with G*Power 3.1 (Heinrich-Heine-University Düsseldorf). Based on a medium effect, a significance level of 5% and a power of 80%, a case number of 78 patients was obtained (two-factor analysis of variance).

Statistical analyses of the results were performed using Graph Pad Prism 9 (La Jolla, USA) and SPSS Version 26 (IBM, Armonk, USA). All data are presented as mean with standard deviation unless indicated otherwise. The existence of a Gaussian distribution of data was evaluated using the Kolmogorov–Smirnov test. The Levene test was applied to test for equal variances. The Mann–Whitney *U* test was used for group comparisons in case of not normally distributed dichotomous data; otherwise, a Student *t* test or Welch test was applied. Statistical differences of rSO2, end-tidal CO_2_ and mean arterial pressure between groups and timepoints were analyzed using two-way-ANOVA and Tukey’s multiple comparison test. The significance level (alpha) was adjusted for multiple testing (*p* = 0·05/n). Associations between variables were assessed using Spearman’s correlation coefficient.

## Results

This prospective observational trial included 78 patients in two groups. Detailed patient characteristics are summarized in Table 1. Both groups did not differ regarding biological sex distribution, patient size, or body mass index (BMI). ASA classification was higher in the older patient group.

**Table 1:** Patient’s characteristics

	Young patients (*n* = 39)	Old patients (*n* = 39)	Statistical significance
Gender (women/men)	16/23 (41/59)	19/20 (49/51)	*p* = 0.495
Age (years)	29 ± 5	68 ± 67	*p* < 0.001
Height (cm)	174 ± 11	172 ± 10	*p* = 0.381
Body weight (kg)	75 ± 17	75 ± 16	*p* = 0.941
Body mass index (kg/m^2^)	24 ± 5	26 ± 7	*p* = 0.413
ASA status (*n*; (%))			
l	17 (44)	5 (13)	
ll	22 (56)	32 (82)	
lll	0 (0)	2 (5)	
lV	0 (0)	0 (0)	

After a constant level of end-tidal CO_2_ had been achieved, rSO_2_ remained constant in the YP group but increased in OP after mild hypoventilation (*YP*: T0, 73 ± 5% vs. T15: 74 ± 4%; *p* = 0.2321/*OP*: T0, 68 ± 5% vs. T15, 71 ± 5%; *p* = 0.0006).

In contrast, mild hyperventilation decreased rSO_2_ significantly in both groups as compared to its initial value at T0 (*YP*: T0, 72 ± 6% vs. T15, 68% ± 6; *p* < 0.0001/*OP*: T0, 67 ± 6% vs. T15, 65 ± 6%; *p* = <0.0001).

Both groups showed a significant change in rSO_2_ after 15 min of hypo- versus hyperventilation (*YP*, 74 ± 4% vs. 68 ± 6%; *p* < 0.001/*OP*, 71 ± 5% vs. 65 ± 6%; *p* < 0.001). The intergroup difference between mild hypo- versus hyperventilation was the same in both age groups (6 ± 3%; *p* = 0.732).

**Fig. 1:**
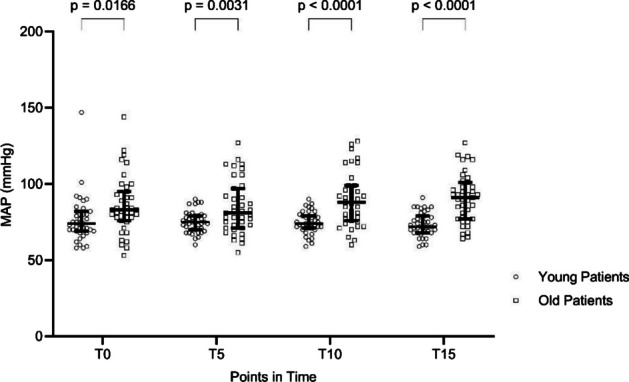
Mean arterial pressure (MAP) during hyperventilation over the course. Median + IQR. T0 = baseline; T5 = 5 min of hyperventilation; T10 = 10 min of hyperventilation; T15 = 15 min of hyperventilation

**Fig. 2:**
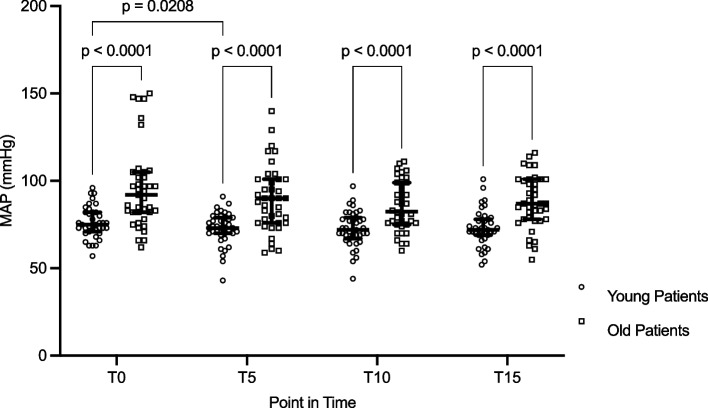
Mean arterial pressure (MAP) during hypoventilation over the course. Median + IQR. T0 = baseline; T5 = 5 min of hypoventilation; T10 = 10 min of hypoventilation; T15 = 15 min of hypoventilation

End-expiratory CO_2_ (etCO_2_) did not differ between age groups at any of the measured points except for T15 after hyperventilation (*p* = 0.0074). PSI values remained unchanged throughout all measurements and were not different between the two age groups (Table 2). The MAP values were higher in the OP-group but remained constant within each group during hypo- and hyperventilation (Figs. 1 and 2, Table 3). In the OP-group, higher doses of norepinephrine were used to achieve pressure targets during hyperventilation (T15: *YP*, 0.016 ± 0.048 mg/h vs. *OP*, 0.199 ± 0.145 mg/h; *p* < 0.001) as well as during hypoventilation (T15: *YP*, 0.015 ± 0.049 mg/h vs. *OP*, 0.203 ± 0.159 mg/h; *p* < 0.001).

**Table 2:** Patient state index values at the different points in time. Data given as mean and standard deviation

Point in Time	Hyperventilation		Hypoventilation		
	Young patients	Old patients	Young patients	Old patients	
T0	30 ±7	32 ± 9	30 ± 8	34 ±11	*p* = 0.0318
T5	30 ± 8	31 ± 10	29 ± 7	32 ± 9	
T10	30 ± 8	31 ± 10	28 ± 7	33 ± 11	*p* = 0.0332
T15	30 ± 8	31 ± 10	28 ± 6	33 ± 10	*p* = 0.0108

*T0* baseline, *T5* 5min of hyperventilation, *T10* 10min of hyperventilation, *T15* 15min of hyperventilation

*PSI* Patient state index

**Table 3:** Mean arterial pressure (MAP) values at the different points in time. Data are given as mean and standard deviation

Point in time	Hyperventilation		Hypoventilation	
	Young patients	Old patients	Young patients	Old patients
T0 (mmHg)	76 ± 15	86 ± 19	76 ± 9	96 ± 24
T5 (mmHg)	75 ± 7	85 ± 18	73 ± 9	90 ± 19
T10 (mmHg)	75 ± 7	90 ± 18	73 ± 10	86 ± 14
T15 (mmHg)	74 ± 8	91 ± 16	73 ± 10	88 ± 15

*T0* baseline, *T5* 5 min of hyperventilation, *T10* 10 min of hyperventilation, *T15* 15 min of hyperventilation

Peripheral oxygen saturation ranged from 94 to 100% independently from the point in time.

## Discussion

The results of this study demonstrate a significant impact of mild hypoventilation and hyperventilation (within the limits of end-tidal normocapnia) on cerebral red blood cell oxygen saturation as determined by NIRS. There was no difference between young and older patients.

NIRS measurements have been used for several years to monitor regional RBC oxygen saturation in the outer cerebral layer, thereby also serving as a surrogate parameter of cerebral perfusion (Bozzani et al. 2022; Milne et al. 2022; Sahinovic et al. 2020). NIRS is consequently used during surgical procedures regularly compromising cerebral blood flow. This includes interventions on the carotid arteries as well as cardiac and thoracic surgeries. According to many study results, implementation of NIRS monitoring is not necessarily associated with a better patient outcome (Yu et al. 2018). Nevertheless, some investigations, especially in the field of cardiac surgery, showed a significant correlation of low intraoperative rSO_2_ values with the occurrence of postoperative cognitive dysfunction (POCD) and postoperative delirium (Ahrens et al. 2023; Momeni et al. 2019; Ortega-Loubon et al. 2019). Other studies failed to demonstrate an association between reduced rSO2 and the incidence of postoperative cerebral complications (Holmgaard et al. 2019). These conflicting study results have been addressed in a recent Cochrane Database analysis, which clearly revealed that the unclear relationship between intraoperative NIRS/rSO_2_ and postoperative outcome is mainly due to insufficient data available to date (Yu et al. 2018).

In addition to the above-mentioned unclear clinical relevance, the NIRS method has a few technical limitations. These relate in particular to the distance between the electrode and the tissue to be measured and the interference with the oxygenated tissue in between (Shaaban-Ali et al. 2021). These aspects must always be considered when examining cerebral oxygenation using NIRS, which is why global statements on cerebral oxygenation must be made with caution.

Hypo- and hypercapnia interfere with cerebral blood flow by changing resistance vessel width (Donnelly et al. 2016; Meng and Gelb 2015; Paulson et al. 1990; Silverman and Petersen 2022). In recent years, studies on the relationship between etCO_2_ and rSO_2_ have been published time and again (Hoffman et al. 2022; Schopfer et al. 2021; Sørensen et al. 2016; Sørensen et al. 2014; Zhang et al. 2020). These consistently showed a correlation between ventilation and rSO2. However, most of the studies were conducted in connection with major surgical procedures involving volume shifts, extracorporeal membrane oxygenation, resuscitation, or in the pediatric field.

A small number of studies have examined the impact of end-tidal CO_2_ on rSO2 via NIRS in healthy subjects. Tisdall and Tachtsidis, as well as Friend et al., were able to show a relationship between hypercapnia and increased rSO_2_ as well as a reduction of saturation during hypocapnia in small study cohorts (*n* = 10, *n* = 15, and *n* = 20) (Friend et al. 2019; Tachtsidis et al. 2009; Tisdall et al. 2009). Kim et al. demonstrated the ability of hypocapnia to reduce rSO_2_ during shoulder surgery in a cohort of 51 patients (Kim et al. 2016). We concentrated on mild hypo- and hyperventilation with end-tidal CO_2_ within the normal physiologic range of 35–45 mmHg whereas in the aforementioned studies end-tidal CO_2_ values < 30 mmHg and > 50 mmHG were used. Low-normal (35 mmHg) end-tidal CO_2_ led to a reduction in rSO2 which proved to be statistically significant in comparison to high-normal (45 mmHg) end-tidal CO_2_ values. This difference is remarkable and has not been described previously. Up to now, it was assumed that changes within the limits of end-tidal CO_2_ of 35–45 mmHg would not have particular consequences regarding manifold parameters, such as regional circulation, vascular resistance, or acid–base balance (Donnelly et al. 2016; Tachtsidis et al. 2009).

Looking at the data obtained in this study, an absolute difference of 6% in regional cerebral RBC saturation might have no clinical impact in healthy subjects. However, one can speculate that in critically ill patients with already reduced rSO_2_ or compromised autoregulation, even minor differences might become clinically relevant. However, to date, there are only a few data available on the limits from which cerebral saturation measured with NIRS influences clinical outcomes. Both absolute and relative individual limits of cerebral saturation are not yet generally accepted.

On the one hand, further data on this aspect would have to follow. On the other hand, standardized collectives as in the field of carotid surgery, heart surgery, or even operations in beach chair position for interventional studies regarding end-tidal CO2 can be imagined here. So far, this is the area in which the most data is available on the correlation between intraoperative cerebral desaturation and postoperative cognitive disorders.

A further aspect is the age-independent effect of ventilation on rSO_2_. The result fits with the few data published so far, which could also show only a minor influence of age on the rSO_2_ (Burkhart et al. 2011). This does not exclude that comorbidities such as arterial hypertension or vascular disorders, which are more common in the elder population, will have an additional impact. However, this needs to be investigated in separate follow-up studies specifically looking at such pathologies.

This study has some limitations. First, it was carried out on healthy patients without relevant cardiocirculatory comorbidities. Nevertheless, this study should serve as a basis for further questions, such as the influence of comorbidities. Here, the singular factor of age should first be investigated alongside the influence of mild hyper- and hypoventilation.

Second, we only evaluated patients undergoing general anesthesia under propofol for maintenance hence excluding inhalation anesthetics with a potential for intrinsic vasoactive properties. On the other hand, a clear strength relates to standardized anesthesia procedures, including close anesthesia depth monitoring by PSI. Third, we did not measure the arterial PaCO_2_ but only the end-tidal CO_2_ as a surrogate of the PaCO_2_. We did this because the removal of arterial CO2 by blood gas analysis was considered too invasive in healthy patients for ethical reasons. Ultimately, it is PaCO_2_ and not etCO_2_ that determines cerebral blood flow. Under physiological conditions, the range between the two parameters is very small. However, under certain conditions, it can vary greatly, especially under controlled ventilation. Nevertheless, we evaluated healthy patients, so that the probability of a large difference between the two values is low.

Nevertheless, this study included a relatively large sample size compared to other studies dealing with this topic.

## Conclusion

Independent of age, even mild hyperventilation within a normal range of values of end-tidal CO_2_ reduced regional rSO_2_ significantly compared to mild hypoventilation.

## Data Availability

Data not available - participant consent.
