# Correction: Impact of mild hypo- and hyperventilation on cerebral oxygen supply during general anesthesia

**DOI:** 10.1186/s13741-026-00658-5

**Published:** 2026-04-30

**Authors:** Philipp Groene, Miriam Rapp, Tobias Ninke, Peter Conzen, Klaus Hofmann‑Kiefer

**Affiliations:** Department of Anaesthesiology, LMU University Hospital, LMU Munich, Marchioninistr. 15, Munich, 81377 Germany

**Correction: Perioper Med 14, 30 (2025)**

**https://doi.org/10.1186/s13741-025-00517-9**

Following the publication of the original article (Groene et al. 2025), a setting error regarding the 2-way-ANOVA was noticed which causes a few *p*-values to change minimally. The conclusion of the study is not altered by the changes.

The sentence “After a constant level of end-tidal CO_2_ had been achieved, rSO_2_ remained constant in the YP group but increased in OP after mild hypoventilation (YP: T0, 73 ± 5% vs T15, 74 ± 4%; *p* = 0.5387 / OP: T0, 68 ± 5% vs. T15, 71 ± 5%; *p* = 0.0032)” should be corrected to: “After a constant level of end-tidal CO_2_ had been achieved, rSO_2_ remained constant in the YP group but increased in OP after mild hypoventilation (YP: T0, 73 ± 5% vs T15, 74 ± 4%; *p* = 0.2321 / OP: T0, 68 ± 5% vs. T15, 71 ± 5%; *p* = 0.0006)”.

The sentence “In contrast, mild hyperventilation decreased rSO_2_ significantly in both groups as compared to its initial value at T0 (YP: T0, 72 ± 6% vs. T15, 68 ± 6%; < 0.0001 / OP: T0, 67 ± 6% vs. T15, 65 ± 6%; *p* = 0.0004)” should be corrected to: “In contrast, mild hyperventilation decreased rSO_2_ significantly in both groups as compared to its initial value at T0 (YP: T0, 72 ± 6% vs. T15, 68 ± 6%; < 0.0001 / OP: T0, 67 ± 6% vs. T15, 65 ± 6%; *p* < 0.0001)”.

The sentence “End-expiratory CO_2_ (etCO_2_) did not differ between age groups at any of the measured points” should be corrected to “End-expiratory CO_2_ (etCO_2_) did not differ between age groups at any of the measured points except for T15 after hyperventilation (*p* = 0.0074).

Table 2 was updated and now shows significant differences between age groups after hypoventilation. Figure 1 was updated, the *p*-values at T0 and T5 changed minimally.

The corrected version of Table 2:

**Table 2:** Patient state index values at the different points in time. Data given as mean and standard deviation

Point in Time	Hyperventilation		Hypoventilation		
	Young patients	Old patients	Young patients	Old patients	
T0	30 ± 7	32 ± 9	30 ± 8	34 ± 11	*p* = 0.0318
T5	30 ± 8	31 ± 10	29 ± 7	32 ± 9	
T10	30 ± 8	31 ± 10	28 ± 7	33 ± 11	*p* = 0.0332
T15	30 ± 8	31 ± 10	28 ± 6	33 ± 10	*p* = 0.0108

T0 = baseline; T5 = 5 min of hyperventilation; T10 = 10 min of hyperventilation; T15 = 15 min of hyperventilation

*PSI* Patient state index

The original version of Table 2:

**Table 2:** Patient state index values at the different points in time. Data given as mean and standard deviation

Point in Time	Hyperventilation		Hypoventilation	
	Young patients	Old patients	Young patients	Old patients
T0	30 ± 7	32 ± 9	30 ± 8	34 ± 11
T5	30 ± 8	31 ± 10	29 ± 7	32 ± 9
T10	30 ± 8	31 ± 10	28 ± 7	33 ± 11
T15	30 ± 8	31 ± 10	28 ± 6	33 ± 10

T0 = baseline; T5 = 5 min of hyperventilation; T10 = 10 min of hyperventilation; T15 = 15 min of hyperventilation

*PSI* Patient state index

The corrected version of Fig. 1:

**Fig. 1:**
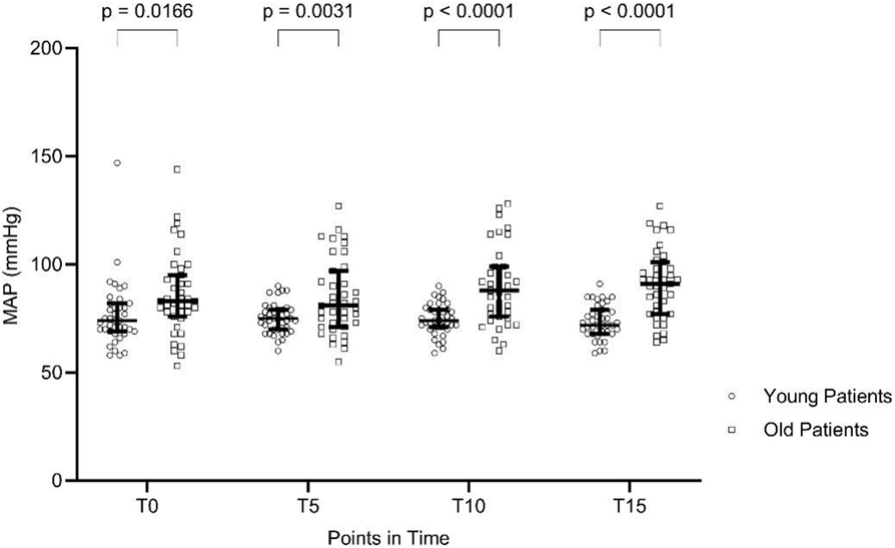
Mean arterial pressure (MAP) during hyperventilation over the course. Median + IQR. T0 = baseline; T5 = 5 min of hyperventilation; T10 = 10 min of hyperventilation; T15 = 15 min of hyperventilation

The old version of Fig. 1:

**Fig. 1:**
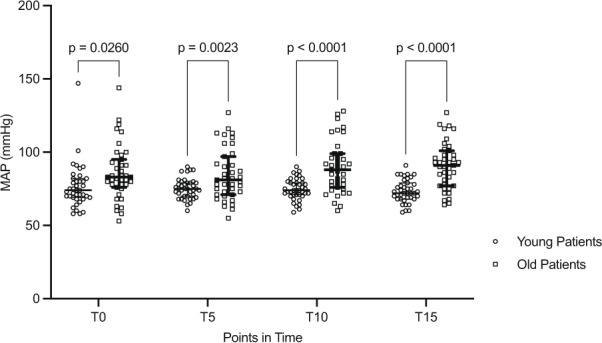
Mean arterial pressure (MAP) during hyperventilation over the course. Median + IQR. T0 = baseline; T5 = 5 min of hyperventilation; T10 = 10 min of hyperventilation; T15 = 15 min of hyperventilation

The original article (Groene et al. 2025) has been updated.
